# Supplementary material for: BlockmiR AONs as Site-Specific Therapeutic MBNL Modulation in Myotonic Dystrophy 2D and 3D Muscle Cells and HSALR Mice
Source: Pharmaceutics. 2023 Mar 31;15(4):1118. doi: 10.3390/pharmaceutics15041118 (PMC10141141; doi:10.3390/pharmaceutics15041118)
Supplement: Supplementary file 1 [file pharmaceutics-15-01118-s001.zip › Supplementary Materials.pdf]

Supplementary Materials: Figures S1–S4

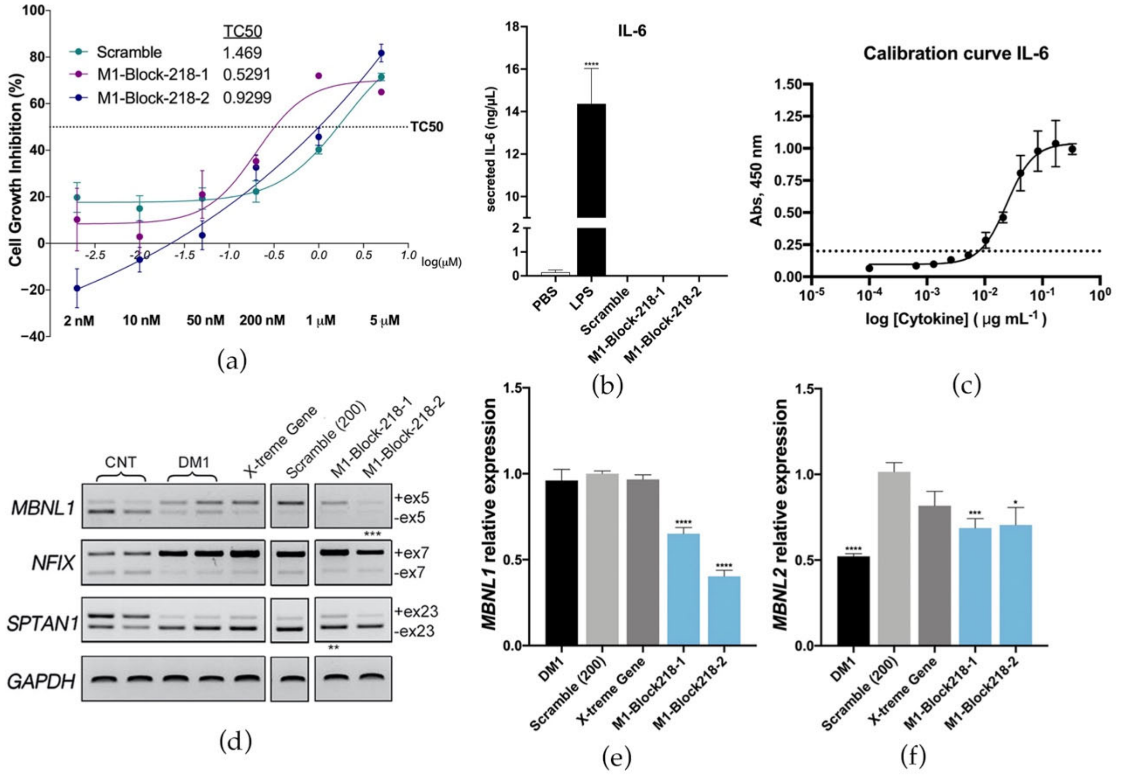

Figure S1. Cell growth inhibition after treatment with blockmiRs. (a) BlockmiRs with miRNA binding sites not confirmed with dual luciferase assay were screened for cell viability at increasing concentrations in DM1 cells. The threshold concentration values at 50% (TC50) for each compound can be seen on the table. Samples were run in quadruplicate. Expression of (b) MBNL1 and (c) MBNL2 transcripts was relatively quantified in quadruplicate after treatment with blockmiRs. MiR-23b targets were transfected at 50 nM while miR-218 targets were transfected at 200 nM. GAPDH was used as an endogenous normalization control and MBNL levels were calibrated with the levels in the respective Scrambles. The DM1 control was calibrated to both Scrambles. All statistical comparisons were performed against the respective Scrambles via Student's t-test. (d) Alternative splicing was analyzed for transcripts MBNL1, NFIX, and SPTAN1 with GAPDH used as an endogenous control. Healthy cells, CNT, and DM1 cells are shown in duplicate as splicing controls. A representative gel is seen above. Amplicons were generated using three different cDNA replicates and averaged for statistical analysis using Image J. All statistical comparisons were performed against the respective Scrambles (50 or 200) via Student's t-test. P-value: $p > 0.05$ (ns), $p \leq 0.05$ (*), $p \leq 0.01$ (**), $p \leq 0.001$ (***), $p \leq 0.0001$ (****). Error bars = SEM. Reproduced/adapted from [29] Universitat de València, 2022.

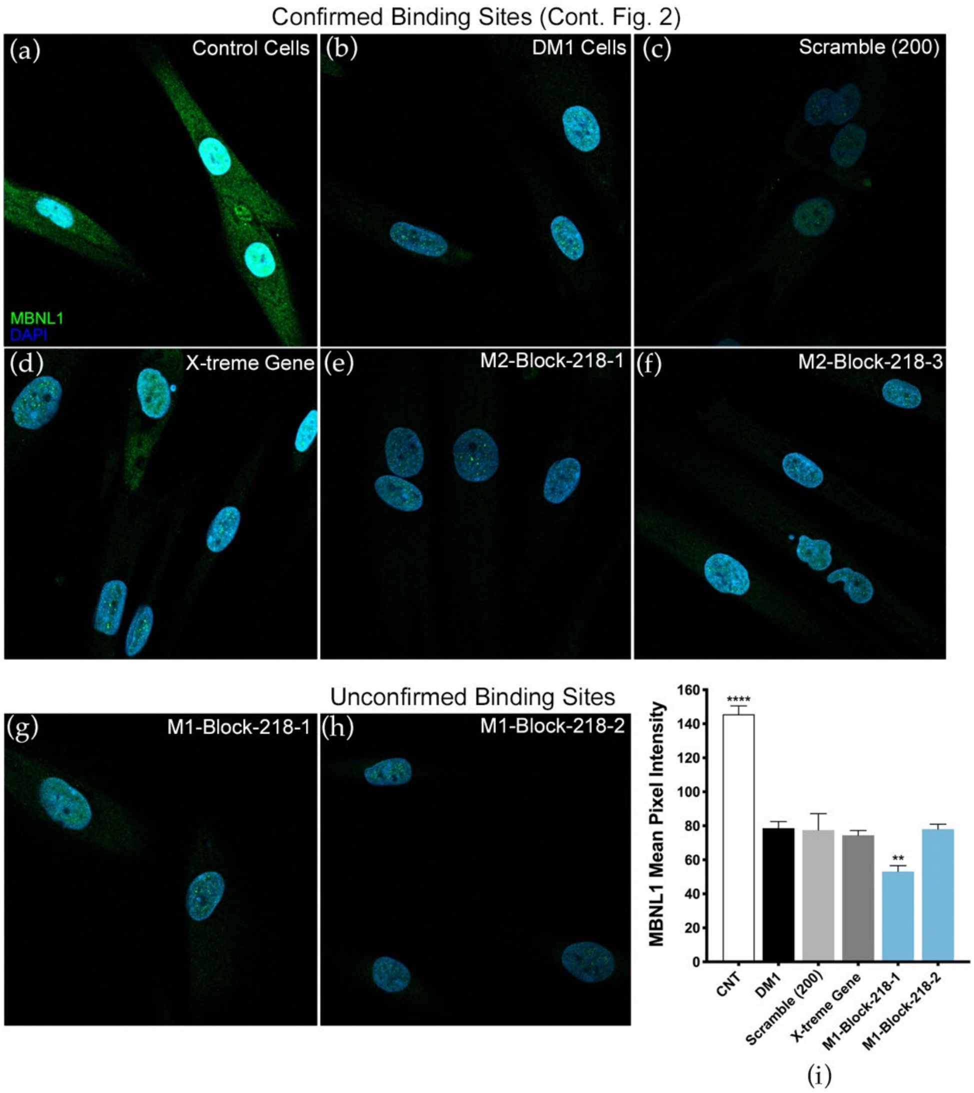

Figure S2. Immunofluorescence of MBNL1 protein for additional blockmiRs. (a-f) Continuation of Figure 3 depicting the immunofluorescence of MBNL1 after treatment with the remaining site-confirmed blockmiRs. DM1 cells were stained for MBNL1 protein (green) and nuclei (DAPI) blue (Cell count: CNT n=63; DM1 n=106; Scramble 200 n=17; X-treme Gene n=119; M2-Block-218-1 n=108; M2-Block-218-3 n=109) (g-h) Immunofluorescence detection of DM1 cells transfected with blockmiRs targeting unconfirmed binding sites. (Cell count: M1-Block-218-1 n=79; M1-Block-218-2 n=91) (i) MBNL1 protein fluorescence was quantified by measuring pixel intensity using Image J with a threshold of 10 and normalized by cell area. All samples compared to Scrambles as a reference via Student's t-test. P-value: $p > 0.05$ (ns), $p \leq 0.05$ (*), $p \leq 0.01$ (**), $p \leq 0.001$ (***), $p \leq 0.0001$ (****). Error bars = SEM. Reproduced/adapted from [29] Universitat de València, 2022.

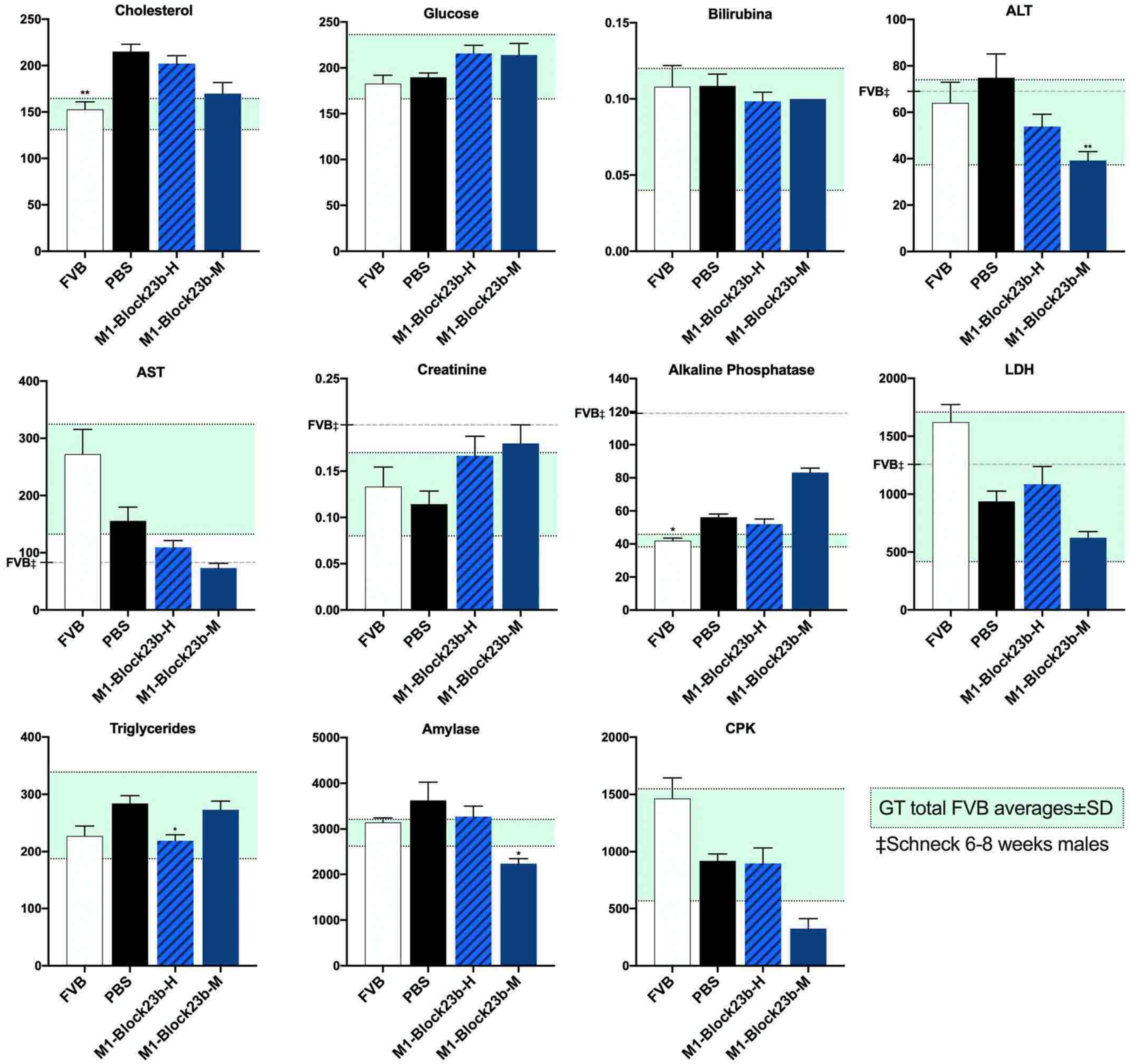

Figure S3. Serum biochemical analysis. Blood serum parameters were analyzed for all treatment groups and compared to empirical PBS controls through Kruskal-Wallis one-way ANOVA. The range of empirical FVB levels are shown in green. Reference FVB levels are shown by dashed grey lines (see legend). P-value: $p > 0.05$ (ns), $p \leq 0.05$ (*), $p \leq 0.01$ (**), $p \leq 0.001$ (***), $p \leq 0.0001$ (****). Error bars = SEM. Reproduced/adapted from [29] Universitat de València, 2022.

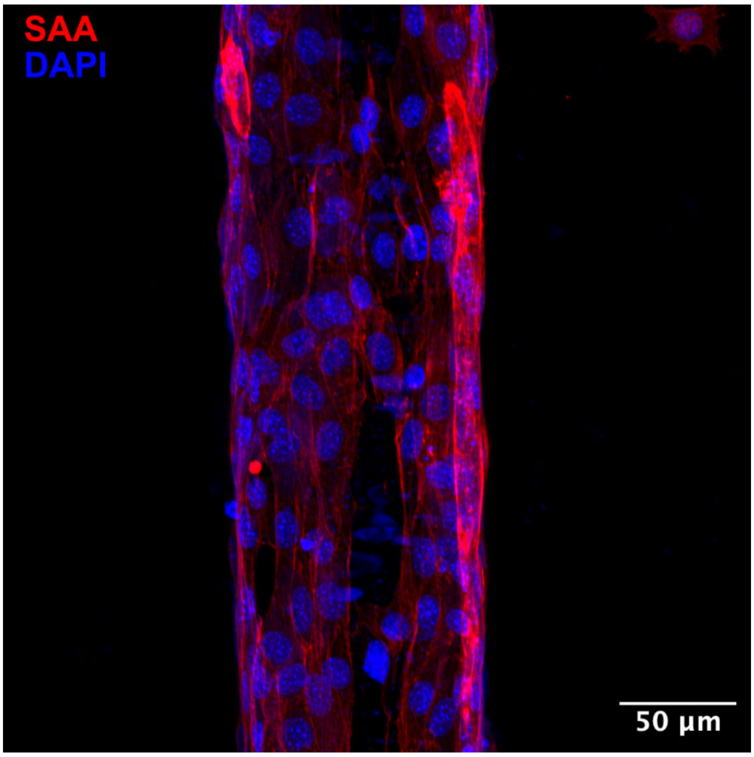

Figure S4. Representative confocal microscopy image of 3D skeletal muscle microtissues stained for the muscle maturation marker sarcomeric α-actinin (SAA, red) and nuclei (DAPI, blue) after 10 days in differentiation conditions.
