# Supplementary material for: BlockmiR AONs as Site-Specific Therapeutic MBNL Modulation in Myotonic Dystrophy 2D and 3D Muscle Cells and HSALR Mice
Source: Pharmaceutics. 2023 Mar 31;15(4):1118. doi: 10.3390/pharmaceutics15041118 (PMC10141141; doi:10.3390/pharmaceutics15041118)
Supplement: Supplementary file 1 [file pharmaceutics-15-01118-s001.zip › SupplementaryMaterial full-length gels.pdf]

Supplementary Materials: Figures S5–S14
Full-length gels and blots

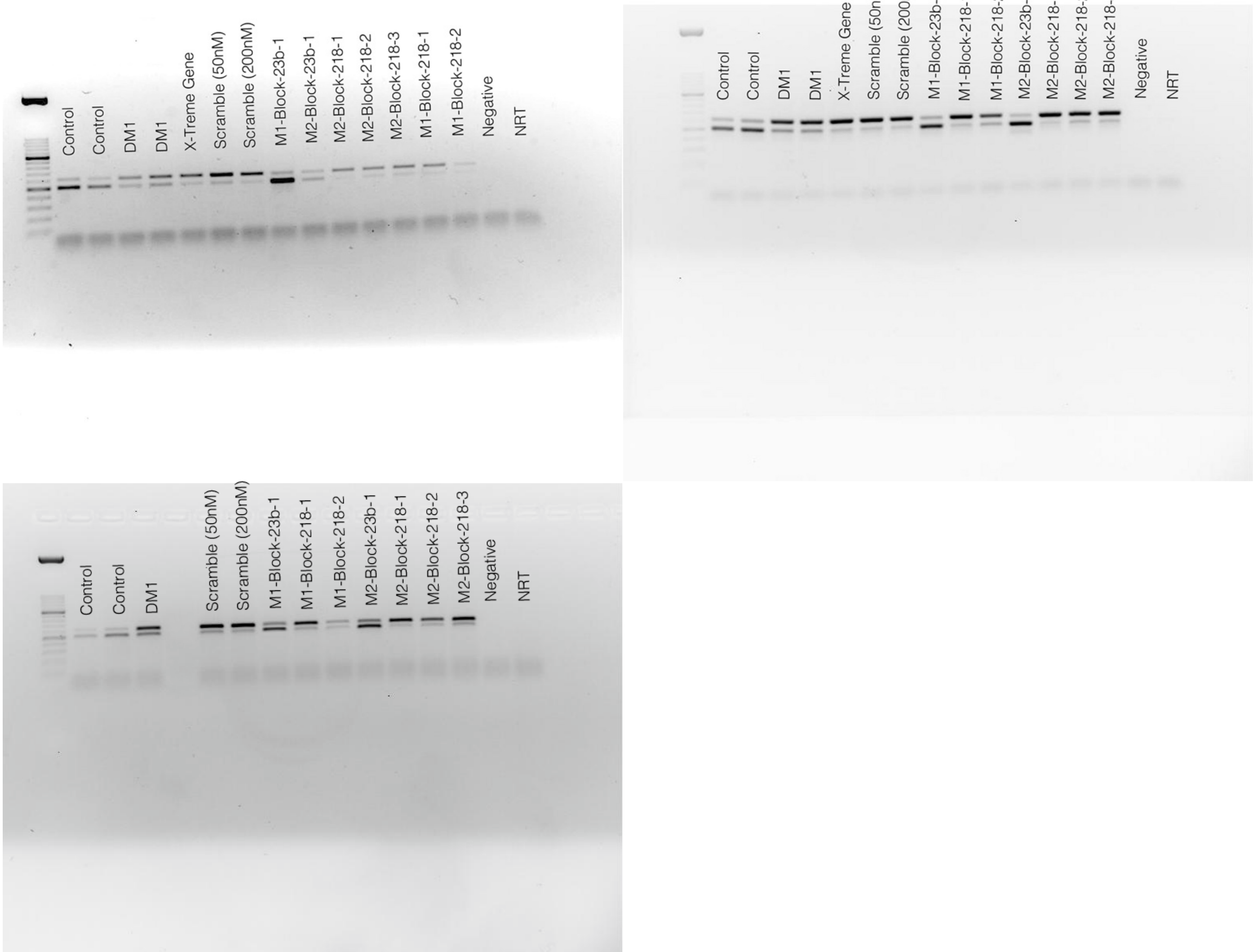

Figure S5. Full-length agarose gels *MBNL1* exon 5 *in vitro*. Amplicons were generated using three different cDNA replicates and averaged for statistical analysis using Image J. See Figure S8 for *GAPDH* endogenous control.

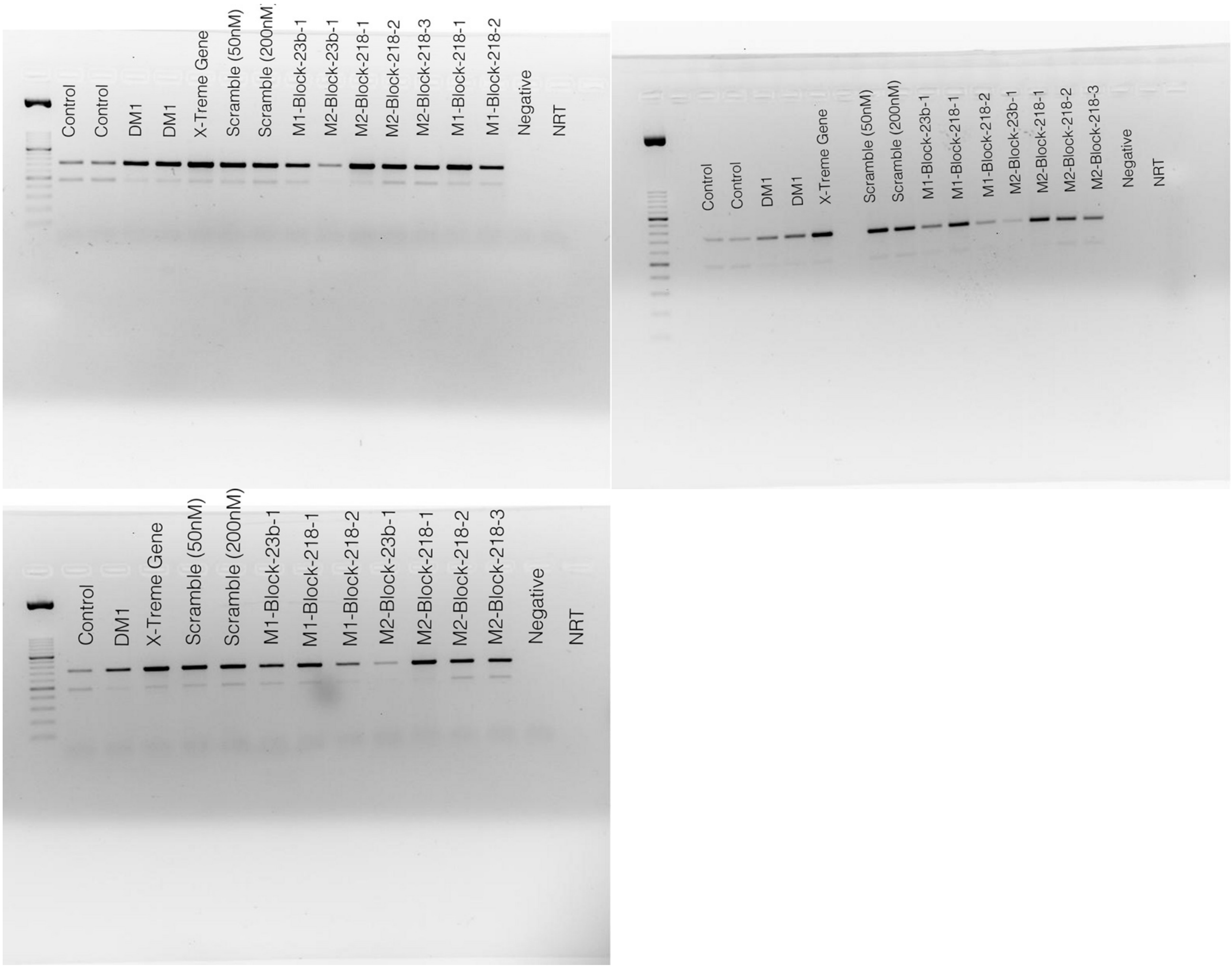

Figure S6. Full-length agarose gels *NFIX* exon 7 *in vitro*. Amplicons were generated using three different cDNA replicates and averaged for statistical analysis using Image J. See Figure S8 for *GAPDH* endogenous control.

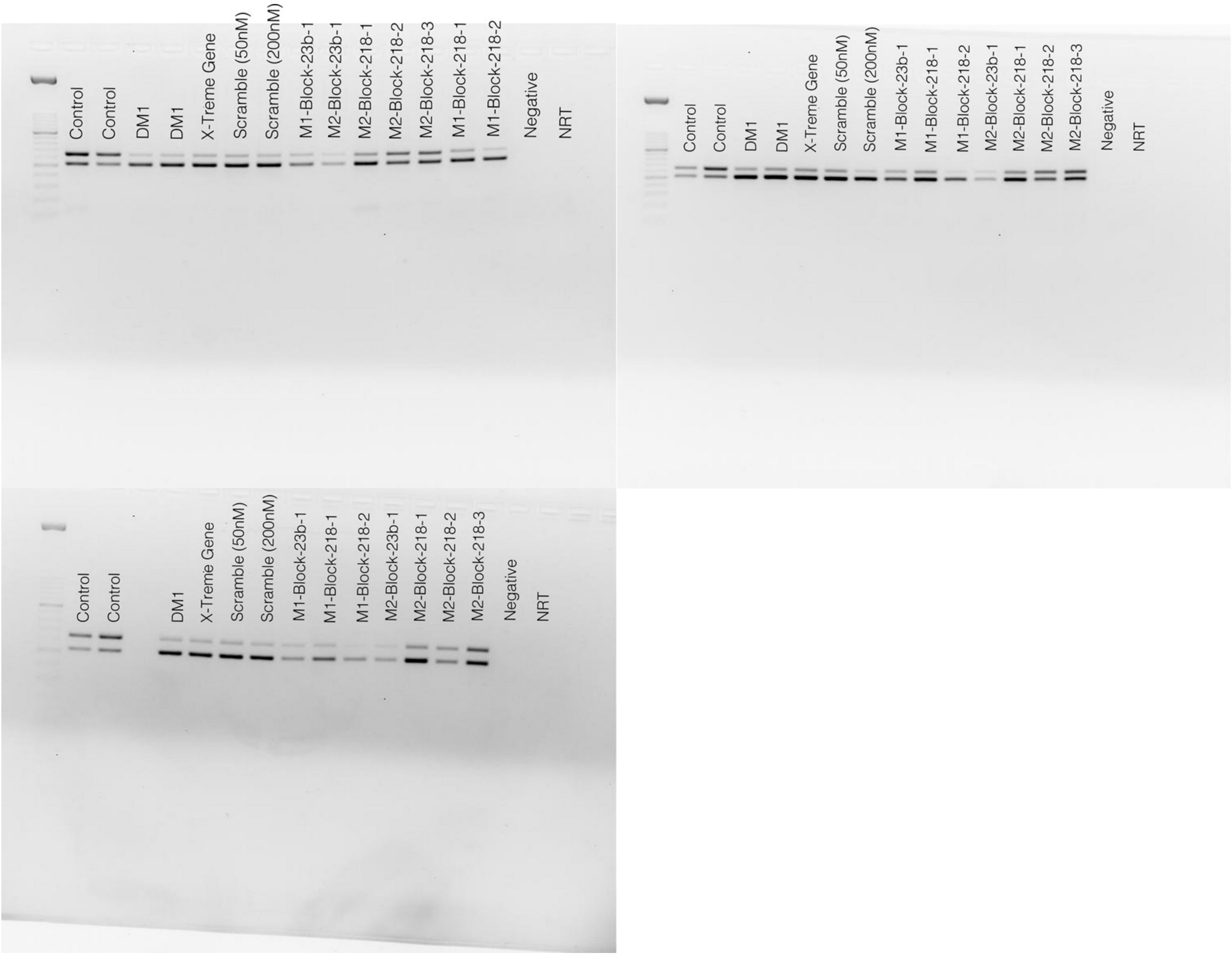

Figure S7. Full-length agarose gels *SPTAN* exon 23 *in vitro*. Amplicons were generated using three different cDNA replicates and averaged for statistical analysis using Image J. See Figure S8 for *GAPDH* endogenous control.

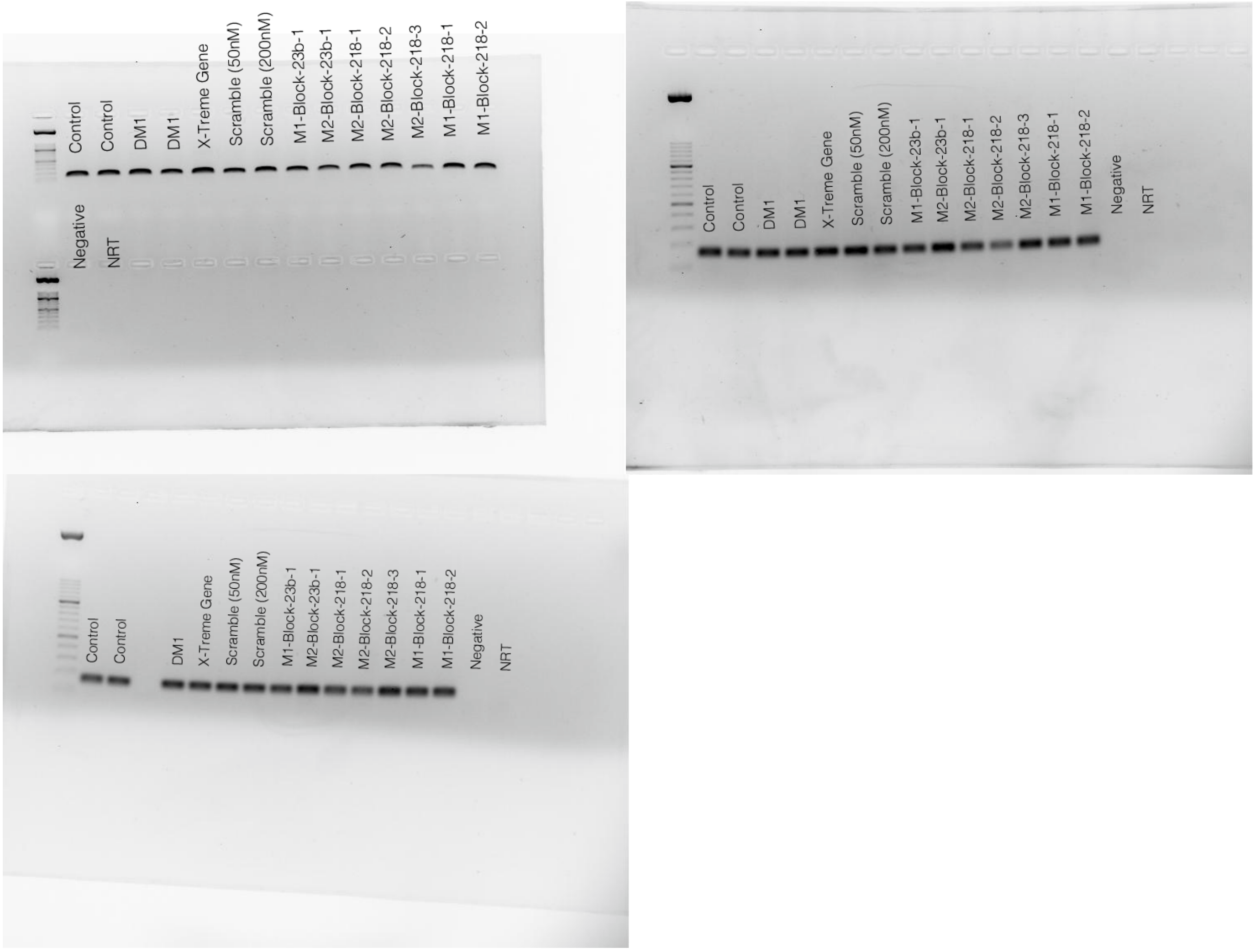

Figure S8. Full-length agarose gels *GAPDH in vitro*. Amplicons were generated using three different cDNA replicates and averaged for statistical analysis using Image J.

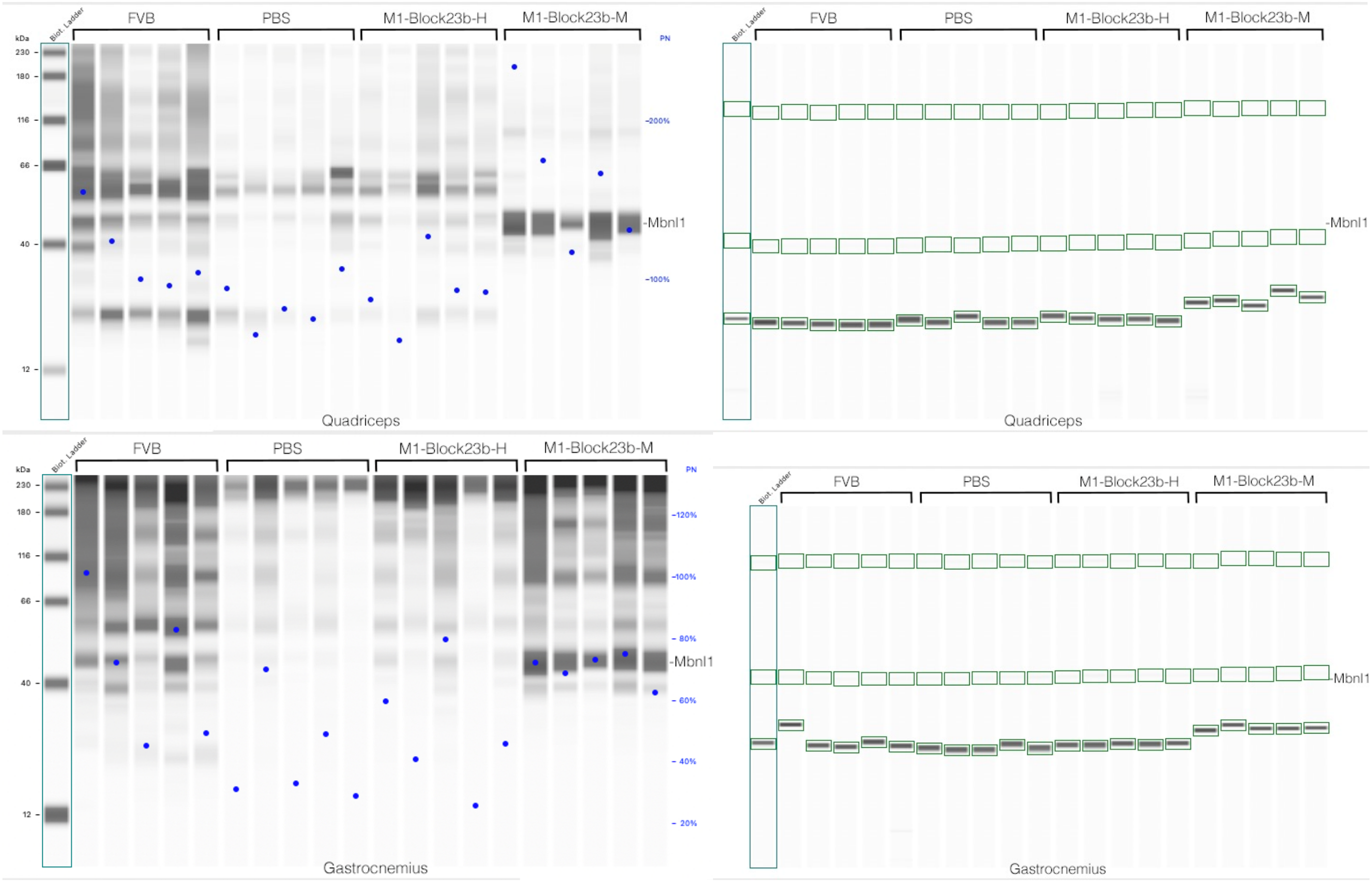

Figure S9. Full-length Jess Simple Western blots. The top two panels are for quadriceps muscle. The bottom two panels are for gastrocnemius muscle. On the left two panels, grey bands represent chemiluminescence readings. Blue dots represent the percentage of total protein used for normalization. On the right two panels, green boxes highlight the three internal standards.

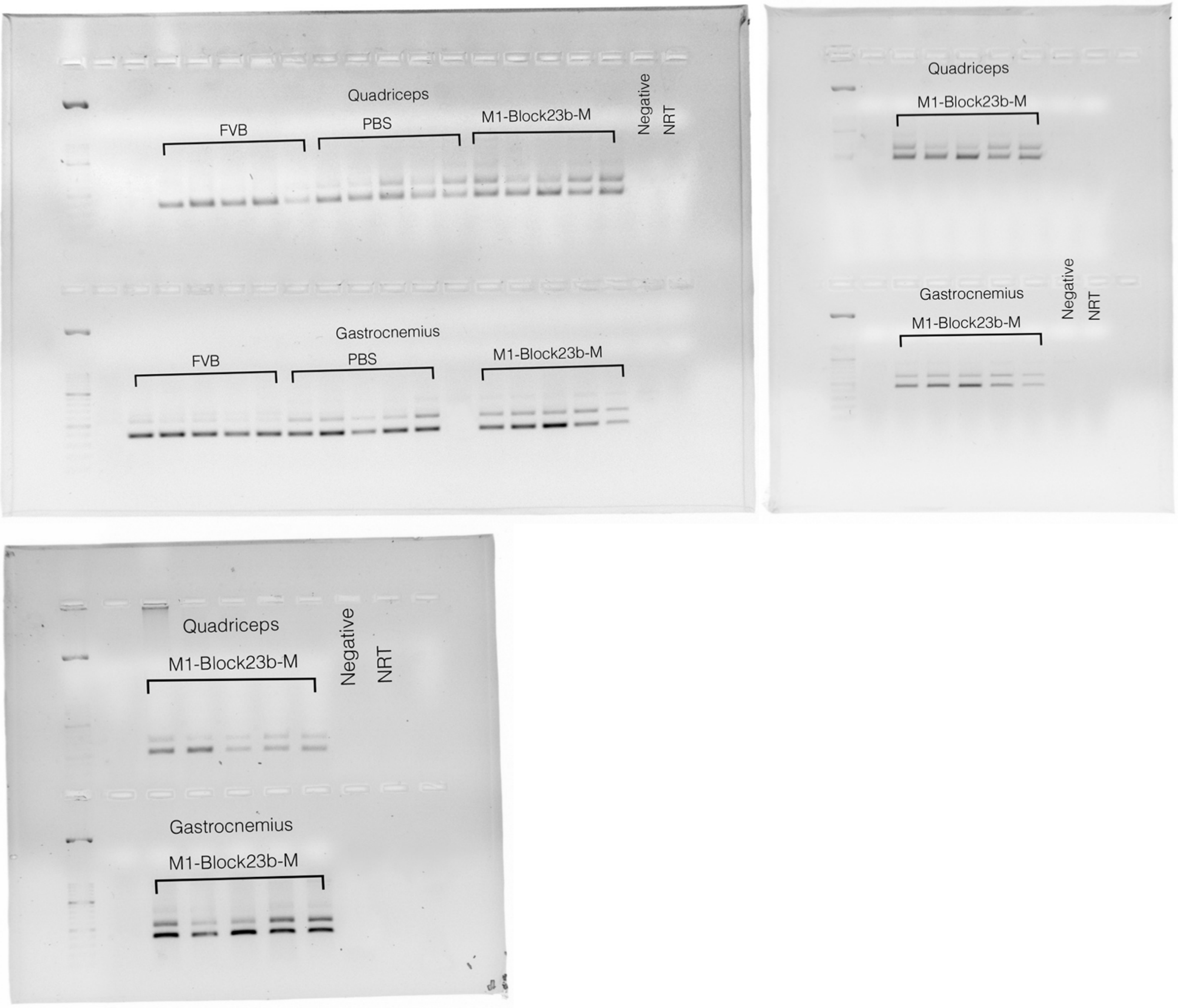

Figure S10. Full-length agarose gels *Clcn1* exon 7a *in vivo*. Amplicons were generated using three different cDNA replicates and averaged for statistical analysis using Image J. See Figure S11 and S14 for *Gapdh* endogenous control.

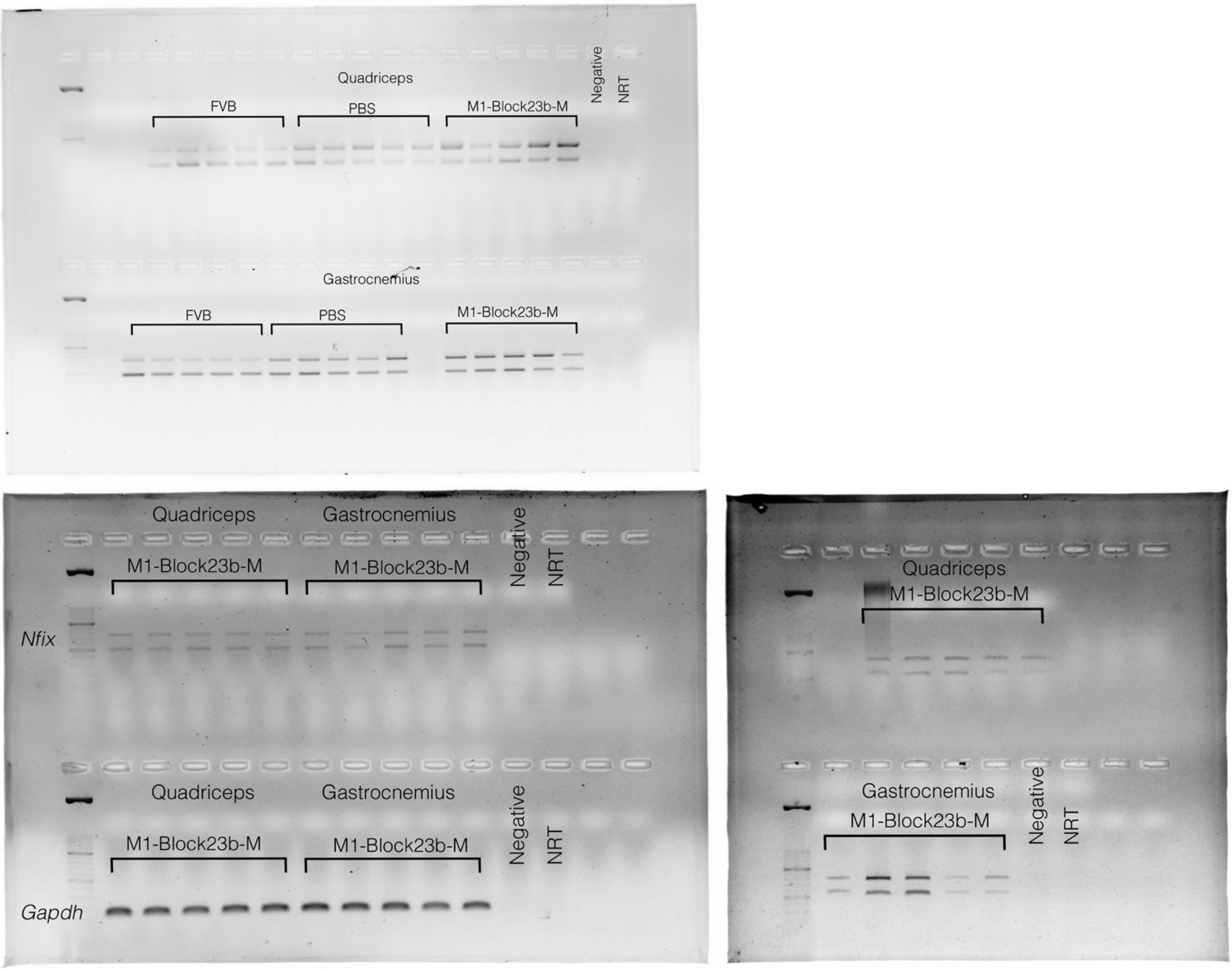

Figure S11. Full-length agarose gels *Nfix* exon 7 *in vivo*. Amplicons were generated using three different cDNA replicates and averaged for statistical analysis using Image J. See Figure S14 for the remaining *Gapdh* endogenous control gels/

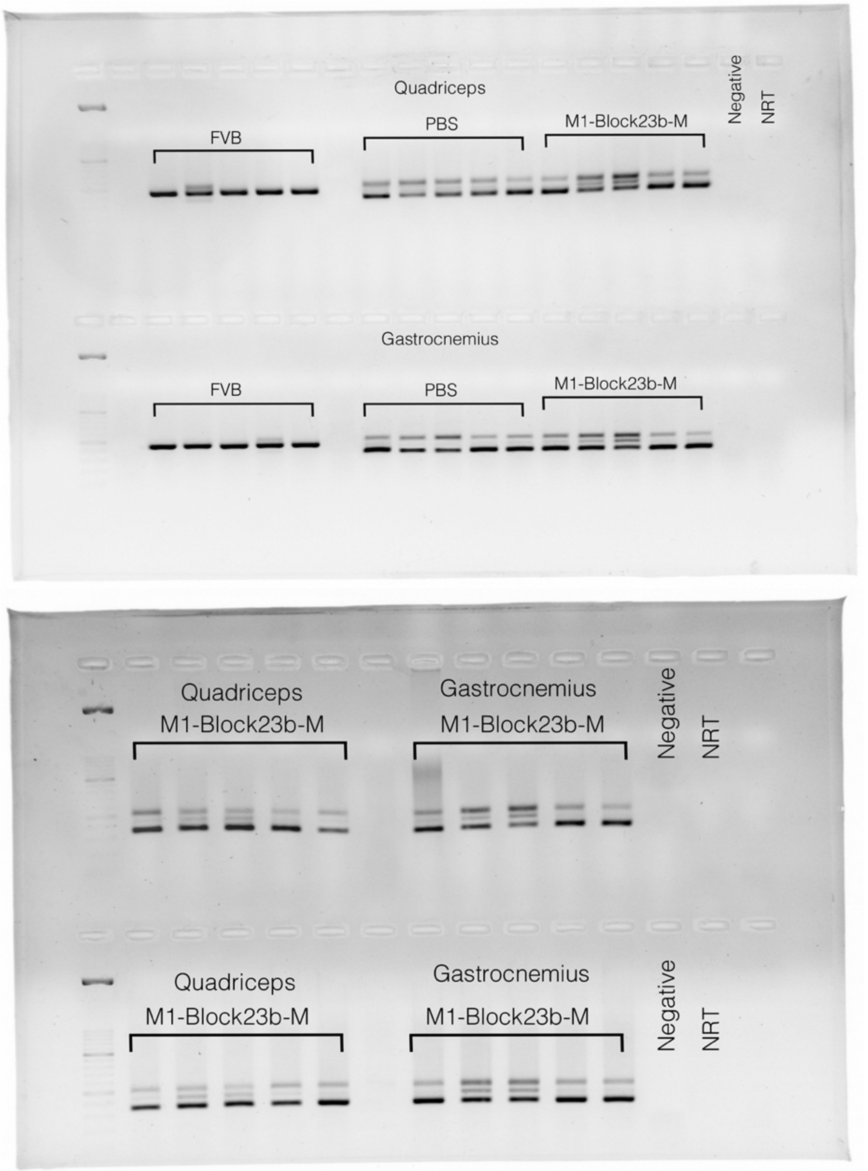

Figure S12. Full-length agarose gels *Atp2a1* exon 22 *in vivo*. Amplicons were generated using three different cDNA replicates and averaged for statistical analysis using Image J. See Figure S11 and S14 for *Gapdh* endogenous control.

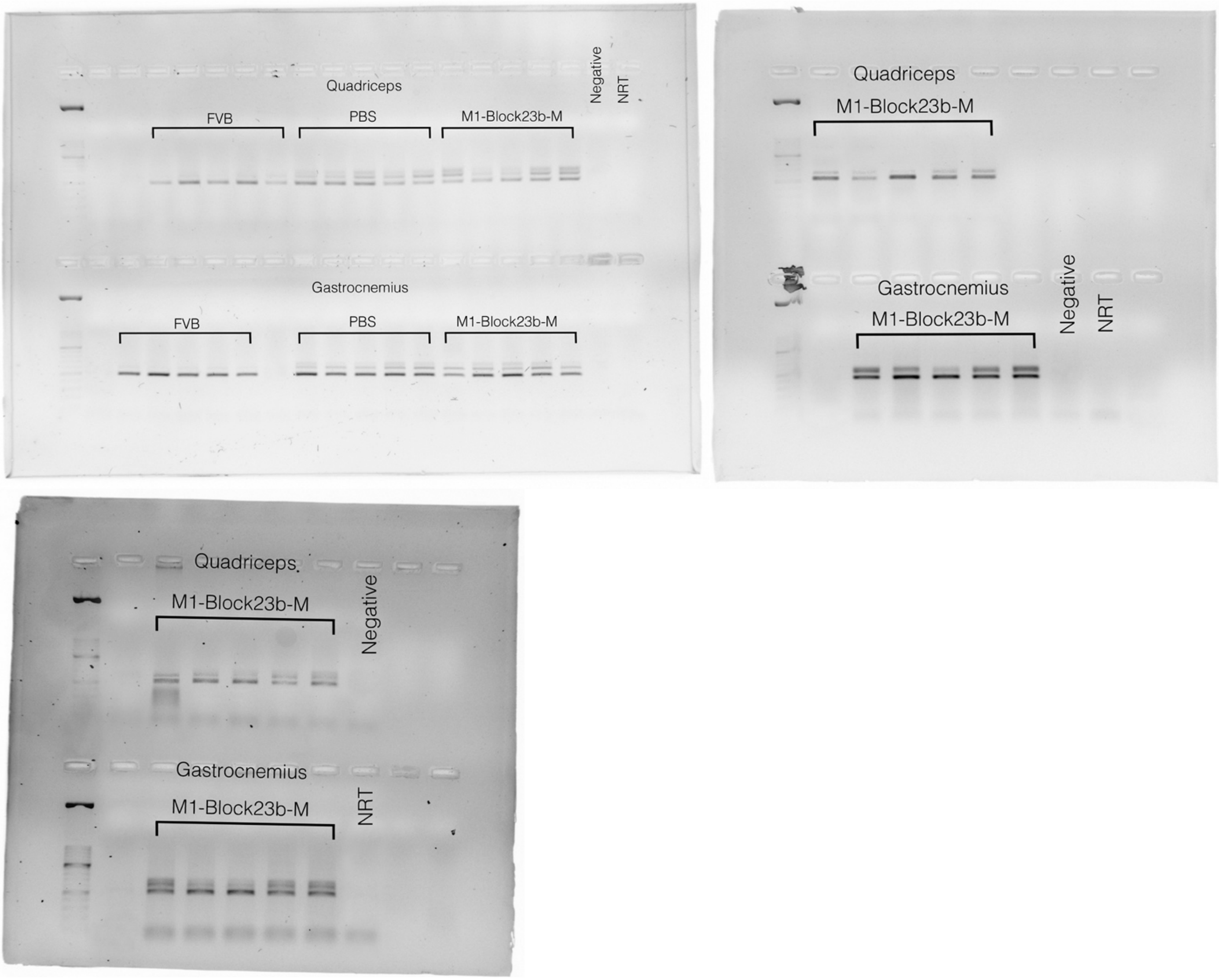

Figure S13. Full-length agarose gels *Mbnl1* exon 5 *in vivo*. Amplicons were generated using three different cDNA replicates and averaged for statistical analysis using Image J. See Figure S11 and S14 for *Gapdh* endogenous control.

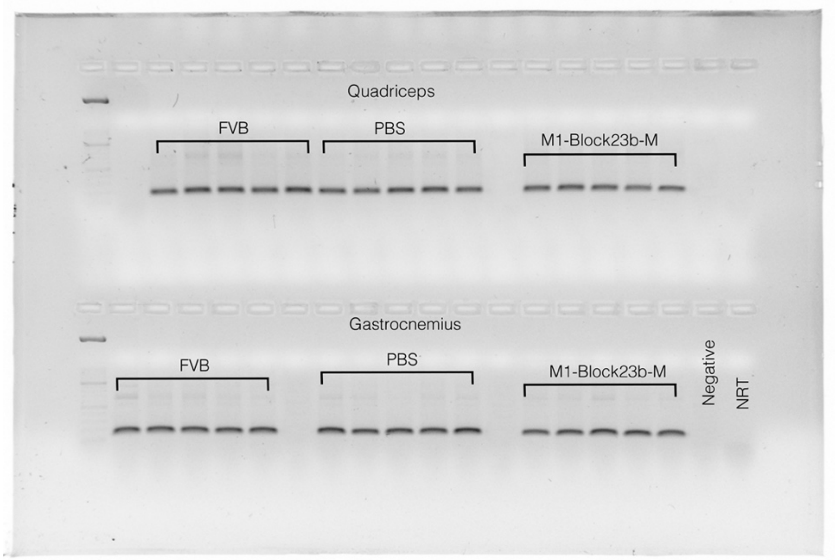

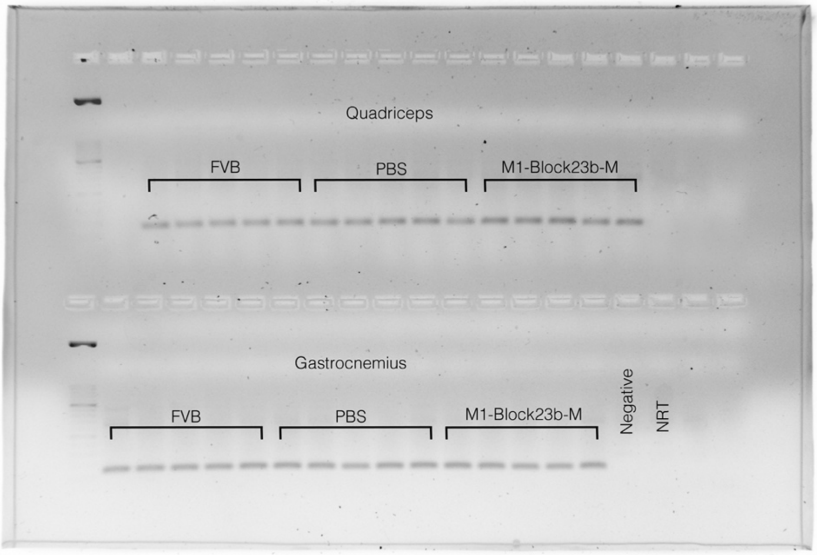

Figure S14. Full-length agarose gels *Gapdh in vivo*. Amplicons were generated using three different cDNA replicates and averaged for statistical analysis using Image J. See Figure S11 for remaining *Gapdh* endogenous control gels.
